# Dimensionality of Carbon Nanomaterials Determines the Binding and Dynamics of Amyloidogenic Peptides: Multiscale Theoretical Simulations

**DOI:** 10.1371/journal.pcbi.1003360

**Published:** 2013-12-05

**Authors:** Nevena Todorova, Adam J. Makarucha, Nicholas D. M. Hine, Arash A. Mostofi, Irene Yarovsky

**Affiliations:** 1 Health Innovations Research Institute, Melbourne, Australia; 2 Department of Materials and the Thomas Young Centre for Theory and Simulation of Materials, Imperial College London, London, United Kingdom; University of Houston, United States of America

## Abstract

Experimental studies have demonstrated that nanoparticles can affect the rate of protein self-assembly, possibly interfering with the development of protein misfolding diseases such as Alzheimer's, Parkinson's and prion disease caused by aggregation and fibril formation of amyloid-prone proteins. We employ classical molecular dynamics simulations and large-scale density functional theory calculations to investigate the effects of nanomaterials on the structure, dynamics and binding of an amyloidogenic peptide apoC-II(60-70). We show that the binding affinity of this peptide to carbonaceous nanomaterials such as C60, nanotubes and graphene decreases with increasing nanoparticle curvature. Strong binding is facilitated by the large contact area available for π-stacking between the aromatic residues of the peptide and the extended surfaces of graphene and the nanotube. The highly curved fullerene surface exhibits reduced efficiency for π-stacking but promotes increased peptide dynamics. We postulate that the increase in conformational dynamics of the amyloid peptide can be unfavorable for the formation of fibril competent structures. In contrast, extended fibril forming peptide conformations are promoted by the nanotube and graphene surfaces which can provide a template for fibril-growth.

## Author Summary

Investigation of the effects of nanomaterials on biological systems is crucial due to the increasing exposure to nanostructured materials with the growing developments and applications of nanotechnology in everyday life. Nanoparticles have been shown to have an effect on protein structure and interfere with protein self-assembly leading to the development of amyloid fibrils responsible for many debilitating diseases, such as Alzheimer's, Parkinson's and prion related diseases. Computational techniques enable investigation of such systems at the atomistic and electronic levels providing insight into properties not available from experiments. We employ a novel combination of computational methods, including large-scale electronic structure calculations and classical molecular dynamics to investigate the behavior of amyloidogenic apoC-II peptide in the presence of carbonaceous nanoparticles, the most prevalent form of nanoparticles found in the environment. Our results showed that carbon nanoparticles have significant effects on the peptide structure, dynamics and binding affinity. Specifically, the dimensionality and curvature of the nanomaterial can either facilitate or hinder their interaction with amyloidogenic peptides and make them adopt conformations capable of inhibiting or promoting fibril growth. These findings are important for rational design of amyloid fibril inhibitors as well as the elucidation of possible toxic effects of carbon based nanomaterials.

## Introduction

The fast-developing field of nanotechnology has already had a significant impact in numerous areas of science and technology due to the ability to control the properties of nanomaterials with greater precision [1]–[3]. Despite the remarkable speed of developments in nanoscience, little is known about the effects of nanomaterials on biological matter [4]. There is a growing concern that nanomaterials, specifically those used for medical applications, may induce cytotoxic effects [5]. In addition, engineered nanomaterials, which are increasingly being used in industry and the manufacture of household goods have the ability to permeate blood-brain barriers and thus have the potential to damage cells *in vivo* [6]. The toxicity of nanoparticles has been associated with fibril formation, where nanoparticles can cause localization of peptides and proteins on their surfaces and promote undesirable aggregation that can favor formation of amyloid fibrils. These highly-structured protein aggregates are responsible for many degenerative diseases such as Alzheimer's, Creutzfeld-Jacob disease, and dialysis-related amyloidosis [7]–[10].

Carbonaceous nanoparticles are one of the most prevalent types of nanomaterials present in the environment. These air-borne particles are continuously injected into the atmosphere in large quantities through the process of combustion and, at the smallest scale, are in the form of clusters with nanometric dimensions. Carbon based nanomaterials, such as fullerenes, nanotubes and graphene surfaces, have been widely studied for potential applications due to their outstanding mechanical, thermal and electronic properties. There is, however, a growing volume of literature that alerts to the potential harm from both intentional (medicinal) and unintentional exposure of living organisms to such particles [6], [11], [12]. Comprehensive understanding of organic-inorganic interactions is crucial in order to minimize the potential toxicological effects associated with advances in the development and use of such nanomaterials [13], [14].

Computational modeling has been used extensively to study the dynamic, thermodynamic and mechanical properties of biological systems. Recent reviews summarize the application of computer simulations to the study of biological matter in the presence of nanomaterials, specifically the common modes by which nanomaterials interact with proteins, DNA and lipid membranes [15]–[19]. Physicochemical properties that may be important in understanding the toxic effects of nanomaterials include particle size and size distribution, shape, exposed surface area, internal structure and surface chemistry [20]. Much research has focused on the characterization of carbon-based nanomaterials such as fullerenes, carbon nanotubes and graphene surfaces [21]–[23]. At the same time, experiments involving carbonaceous nanomaterials in biological milieu are still limited and the interactions involved are not well understood [24]. Specifically, there are some contrasting findings that have recently been published on the role of carbon nanotubes in fibril formation. Linse et al. found an increase in the rate of fibrillation by β2-microglobulin in the presence of carbon nanotubes, where they suggested that a locally increased concentration of protein on the carbon nanotubes surface promotes oligomer formation [9]. Two other separate studies also suggested that carbon nanotubes act as catalyst for fibril formation [25], [26]. In contrast, Ghule et al. found that multi-walled carbon nanotubes inhibit amyloid aggregation of the human growth factor protein, hFGF-1 by encapsulating the protein structure and suppressing like-protein interactions [27]. Furthermore, recent computational studies of Aβ peptides found that carbon nanotubes drive the formation of β-barrels around the nanoparticle [28], [29]. The authors suggested that this type of aggregation would lead to; 1) blocking of the peptide structure for further peptide association; 2) reducing the population of monomers/oligomers available for fibril growth; and thus resulting in an inhibition of Aβ fibrillation [28]. In addition, they proposed that the hydrophobic and π-π interactions between the Aβ peptide and carbon nanotube inhibit β-sheet formation and destabilize fibril-seeds into random coil aggregates, which would increase the nucleation lag-time and possibly reverse the fibrillation process [29]. It is evident from the works presented above that there are contrasting views on the role of carbon nanomaterials in fibril formation. However, there is substantial evidence that suggests carbon nanomaterials can have fibril inducing and inhibiting capabilities depending on the structural architecture of the nanoparticle itself and more importantly, the affinity of the peptide/protein under investigation, which plays a crucial role in the propensity for aggregation and/or fibril formation on nanoparticles [18], [19].

While advances in experimental techniques are able to probe ever-smaller length-scales and ever-shorter timescales, atomistic modeling is a valuable complementary approach for a systematic investigation of detailed mechanisms of nanoscale phenomena at the atomistic and electronic levels [23], [30]–[40]. Herein, we present a computational study investigating the effects of curvature and shape of carbonaceous nanomaterials on the structure, dynamics and binding of an amyloidogenic apolipoprotein C-II (apoC-II) derived peptide, apoC-II(60-70). ApoC-II is a 79 amino acid protein, with an important role in lipid transport [41], [42]. Under lipid-depleted conditions, apoC-II readily forms homogeneous fibrils with a “twisted ribbon” morphology and all of the characteristics of amyloid fibrils [43]. ApoC-II amyloid fibrils are commonly associated with atherosclerotic plaques, where they have been found to co-localize with other apolipoproteins and initiate early events in heart disease [44]. Studies have shown that airway exposure to concentrated ambient particles and single-wall carbon nanotubes can promote progression of the atherosclerosis process in apolipoprotein-E knockout mice that develop plaques in blood vessels at early age [45], [46]. Similarly, a study by Vesterdal *et al.* demonstrated that intraperitoneal administration of pristine C60 fullerenes is associated with a moderate decrease in the vascular function of mice with atherosclerosis [47].

The apoC-II peptide derivative, apoC-II(60-70), was found to have the ability to form amyloid fibrils independently [48]. This peptide has been extensively investigated under a range of conditions and in different environments using experimental and computational techniques [33], [34], [37], [38], [49], [50]. Our previous studies using molecular dynamics simulations of the monomeric wild-type apoC-II(60-70) peptide showed that it preferentially adopts hairpin-like structures in solution. This structure was defined as an intermediate state on-pathway for the formation of fibril-seeds. Increased solvent accessible surface area and the relative orientation of the aromatic side-chains were features identified as fibril-favoring for this peptide, as they promoted hydrophobic interactions with other like-peptides. In contrast, increased flexibility and the broader distribution of angles between the aromatic residues of mutated apoC-II(60-70) resulted in slower aggregation kinetics, in other words these features were fibril-inhibiting, as demonstrated by our experiments [34], [37]. Furthermore, our research on oligomeric apoC-II(60-70) showed that extended β-sheet structures stabilize preformed dimers and tetramers of apoC-II(60-70). The results suggested that a tetrameric oligomer in anti-parallel configuration can serve as a possible seed for fibril formation of apoC-II(60-70), where side-chain-side-chain contacts contribute to the fibril stability, while the maximum exposure capacity of the whole peptide (backbone and aromatic side-chains) promotes the growth of the fibril-seed due to the increase of exposure to other peptides [34], [37]. Overall, the solution based studies on the behavior of apoC-II(60-70) in different environments provide benchmarking data for identifying the effects of nanomaterials on the structure and dynamics of this amyloidogenic peptide.

Here, we investigate the behavior of apoC-II(60-70) in the presence of three carbonaceous nanomaterials: a spherical C60 fullerene, a tubular single-wall carbon nanotube and a flat graphene surface. We study the peptide's structure, dynamics and binding, all of which can influence its fibril formation capacity and compare the results with the previously characterized peptide behavior in solution [33], [34], [37], [38]. We apply a novel combination of computational methods, including large-scale electronic structure calculations and classical all-atom molecular dynamics. This approach was recently applied for the first time to investigate the fibril inhibition mechanisms of cyclic apoC-II(60-70) and its linear analogue [39]. This combined modeling approach enables investigation of the fundamental driving forces behind the interactions of the peptide with nanomaterials and their effects on the peptide structure, dynamics and binding affinity.

## Methods

### Atomistic simulations of peptide-nanomaterial systems

To investigate the effects of carbonaceous nanomaterials on the structure and dynamics of apoC-II(60-70) (MSTYTGIFTDQ, 169 atoms) a series of simulations were performed with different starting peptide conformations and arrangements. The fullerene particle consisted of 60 carbon atoms with a radius of ∼3.5 Å. The nanotube was modeled as a (5,5) single-walled tube with 320 atoms in an open ended armchair arrangement, 6.78 Å in diameter and ∼38 Å in length, which was sufficiently long to prevent interactions between the peptide and the edges. Graphene was modeled as a periodic single sheet of 2160 carbon atoms in a hexagonal arrangement to represent an infinite graphene surface. The initial configurations were constructed by positioning the peptide 4.5–10 Å from the nanomaterial (see Table S1 and S2 in Supporting Information). The peptide together with the nanomaterial was then placed in a periodic simulation cell of at least 60 Å×60 Å×60 Å in dimension.

The molecular dynamics (MD) simulations were performed using the Gromacs 3.3 [51] simulation package, with the interactions between the particles in the system described by the united-atom Gromos forcefield and the 43A1 parameter set. The carbonaceous nanomaterials were modeled using the aromatic sp^2^ carbon parameters. We note that polarizable forcefields which describe the electrostatic interactions with the use of distributed multipoles [32], [40], [52] have been under development for graphitic structures, however, recent studies have shown that classical forcefields produce results comparable to experiment [16], [53], [54].

The Lennard-Jones interactions were truncated at 10 Å, with the long-range electrostatic interactions accounted for by the Particle Mesh Ewald (PME) method [55]. The LINCS algorithm was used to constrain the bond lengths to their equilibrium values [56], enabling a timestep of 2 fs to be applied for all simulations. The VMD software package was used for visualization of the dynamics and analysis of the molecular trajectories [57].

*In vacuo* energy minimization using steepest descent algorithm was initially performed on the peptide-nanoparticle systems to remove steric clashes. The optimized system was then solvated using the SPC water model [58] at a water density of ∼1 g/cm^3^. To neutralize the overall negative charge of the system, an unrestrained counterion (Na+) was included in the simulation cell. Energy minimization on the solvated system was performed to relax all of the atomic degrees of freedom. Subsequently, MD was conducted to allow the solvent to equilibrate around the solutes by keeping the peptide and nanomaterial restrained. A constant pressure of 1 bar and temperature of 300 K were maintained using the Berendsen barostat and thermostat [59]. In all simulations the geometry of the nanomaterial was restrained for ease of monitoring the peptide dynamics.

Two initial non-fibrillar conformations (native and helical) of apoC-II(60-70) peptide were simulated in the presence of each nanomaterial. To further enhance conformational sampling simulations were repeated six times with different starting orientations of apoC-II(60-70) with respect to the nanomaterial, yielding a total 800 ns of data per nanomaterial-peptide complex. The behavior and structures observed in each system exhibited distinctive trends, therefore the results from representative simulations are shown.

### Potential of mean force calculations

Using umbrella sampling together with the weighted histogram analysis method (WHAM) [60], potential of mean force (PMF) profiles were generated to evaluate the free energy of dissociation (*ΔG*) for apoC-II(60-70) bound to each nanomaterial in solution. This method was applied to explicitly solvated systems and therefore accounts for the entropic contributions in the determination of the dissociation energies. We determined the PMF as a function of separation distance between the center of mass of the nanomaterial and the α-carbon of the glycine residue in apoC-II(60-70). To acquire the PMF profiles, a series of simulations (windows) were performed at increasing distance between the peptide and nanomaterial, starting from typical equilibrium structures of the peptide-nanomaterial complex. The peptide was restrained at each window using Hookean functions with a force constant of 8000 kJmol^−1^ nm^−2^. In the present work, *ΔG* and PMF both refer to the free energy required to bring the peptide and nanomaterial from an associated form, which defines our zero of free energy, to some separation *d*. Adjacent windows were separated by 0.5 Å and each window was simulated for 15 ns with at least 30 windows used (until the peptide was fully dissociated from the nanomaterial), resulting in a total simulation time of at least 450 ns per nanomaterial complex. WHAM was subsequently applied on the final 5 ns of simulations to remove the biasing potentials and obtain the unbiased PMF profiles. The overlap between neighboring windows was monitored to ensure the suitability of the selected spring constant and sufficient conformational sampling (not shown).

### Electronic structure and binding energy calculations

Binding energy calculations were performed on multiple structures selected from the classical forcefield simulations of each peptide-nanomaterial system (more details in aromatic tracking section). Based on the findings of a recent methodological study, classical energy minimization was performed in solution prior to electronic structure calculations [61]. This procedure reduced any electrostatic artefacts that may arise due to the electronic structure calculations being performed in vacuum while retaining the major structural features of the system obtained during the fully solvated MD simulations. Single point electronic energy calculations performed on the resultant frames were used to calculate *in vacuo* binding energies between the peptide and nanomaterial. We determined the binding energy (*E_b_*) of apoC-II(60-70) peptide on each nanomaterial, as
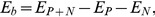
(1)where *E_P+N_* is the total energy for the peptide-nanomaterial complex, *E_P_* is the total energy of the apoC-II(60-70) peptide and *E_N_* is the total energy of the isolated nanomaterial.

The linear-scaling DFT code ONETEP [62] was used, which combines linear scaling computational efficiency with accuracy that is comparable to traditional plane-wave DFT codes. Such efficiency opens up the possibility of performing accurate DFT calculations on thousands and tens of thousands of atoms, including proteins [63]–[65] and various nanomaterials [66], [67]. ONETEP achieves linear scaling by exploiting the ‘near-sightedness’ of the single-particle density matrix *p*(**r**,**r'**) in non-metallic systems,
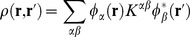
(2)where **K** is the density kernel and *φ*_α_ are a set of strictly localized non-orthogonal generalized Wannier functions (NGWFs) [68]. The total energy is self-consistently minimized with respect to both the density kernel and the NGWFs. The NGWFs are expanded in a basis set of periodic sinc (psinc) functions [69], which are equivalent to a plane-wave basis, and are optimized *in situ*, giving plane-wave accuracy and allowing the accuracy to be systematically improved with a single kinetic energy cut-off parameter.

The PBE generalized-gradient approximation was used to describe exchange and correlation [70], and norm-conserving pseudopotentials were employed to describe the interactions between electrons and nuclei. Dispersion interactions were accounted for using a DFT+D approach [71]. Dispersion-corrected DFT has been shown to produce accurate results for weakly interacting systems, such as aromatic composites [72] and protein-ligand complexes [73]. The supercell dimensions for each system were sufficiently large to prevent interactions between periodic images. In all cases, NGWF radii of 8 bohr were used for all atoms, no truncation was applied to the density kernel, the kinetic energy cut-off for the psinc basis was 880 eV, and the Brillouin zone was sampled at the Γ-point only.

### Aromatic tracking

**Figure 1:**
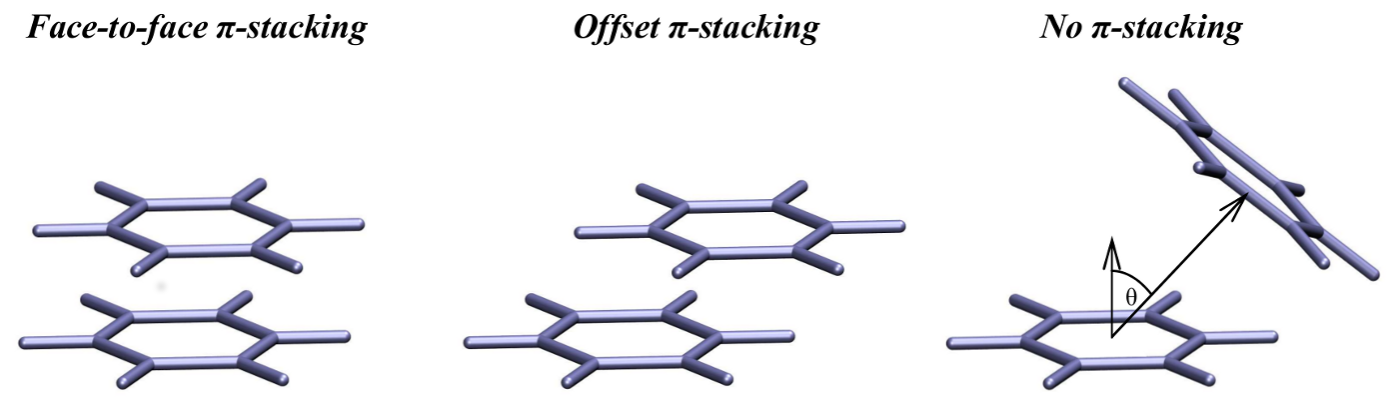
Aromatic ring arrangement categories. Phenyl rings demonstrating the aromatic ring arrangement categorized in three groups: face-to-face π-stacking, offset π-stacking and no π-stacking. To help in the interpretation of the cut-offs applied to categorize each aromatic arrangement, only the angle between the plane normal of the rings is shown (image on the right), while all other distance cut-offs are pair-wise in nature.

The role of aromatic residues in the adsorption of apoC-II(60-70) to each nanomaterial was investigated by tracking the placement of the peptide's aromatic rings across each graphitic surface. This technique determines the position and orientation of the aromatic rings in amino acids relative to the rings within the nanomaterials' surface at every step of the MD trajectories. The aromatic ring arrangement was categorized into three groups: no π-stacking, offset π-stacking and face-to-face π-stacking (Figure 1). The criteria to determine no π-stack register were a pair-wise contact distance over 4.5 Å between any two atoms of the aromatic ring and nanomaterial; or an angle greater than 30° between the plane normal of the aromatic ring and nanomaterial surface [74]. Face-to-face π-stacking was accounted for when the displacement between the centroids of the phenyl rings of the aromatic residues and centroid of the nearest hexagonal carbon ring was less than 0.71 Å (half the carbon-carbon bond length). A displacement greater than 0.71 Å was considered as offset π-stacking. ApoC-II(60-70) peptide having two aromatic rings in its sequence (Tyr63 and Phe67) resulted in six possible ring arrangements relative to the nanomaterials surface. The categories were defined as: (1) no π-stacking by both rings; (2) offset π-stacking by one ring and no π-stacking by the other; (3) offset π-stacking by both rings; (4) face-to-face π-stacking by one ring and no π-stacking by the other; (5) face-to-face π-stacking by one ring and offset π-stacking by the other; (6) face-to-face π-stacking by both rings. Once the aromatic arrangement was categorized, each group underwent structural clustering with RMSD cut-off of 2 Å for the entire peptide using the single linkage clustering method to determine the most frequently sampled structure within each π-stacking category. Three representative structures from each π-stacking group were selected and underwent electronic structure calculations to determine their binding energies.

## Results/Discussion

Explicitly solvated molecular dynamics simulations were used to characterize the interactions between the amyloidogenic peptide apoC-II(60-70) and three exemplar carbonaceous nanomaterials. ApoC-II(60-70) showed a strong affinity to the nanomaterials, where the peptide came in contact with the nanomaterial within the first 20 ns of simulation and remained adsorbed for the entire trajectory. Below we analyze the bound states and the mechanisms responsible for the binding.

### Secondary structure evolution and interactions

**Figure 2:**
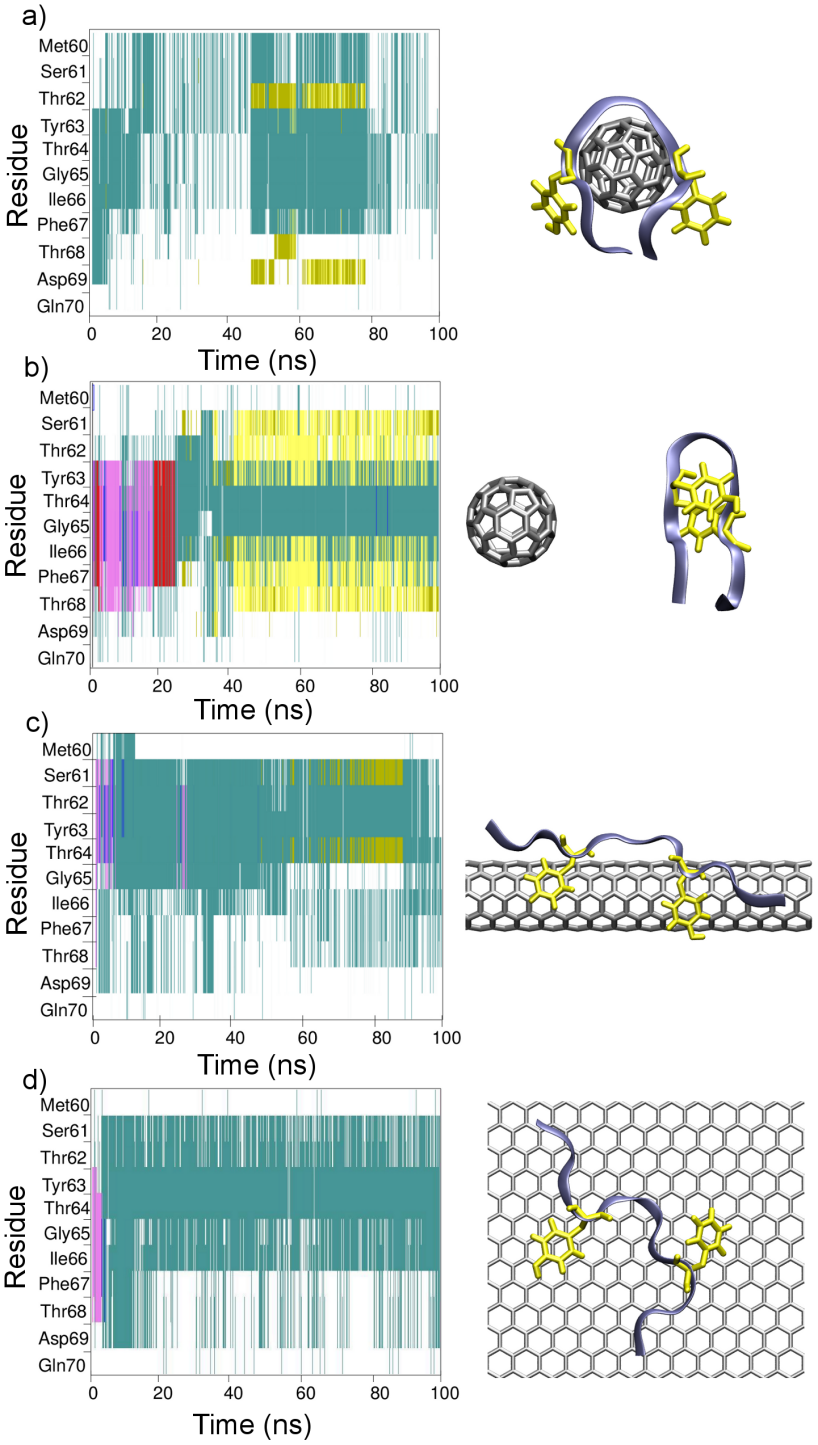
Secondary structure evolution plots for typical behaviors observed of apoC-II(60-70) in the presence of nanomaterials. (a and b) C60, (c) nanotube and (d) graphene surface; Secondary structure color codes: magenta = α-helix; red = π-helix; cyan = turn; white = coil; yellow = extended conformation; green = hydrogen bridge. Screen shots depicting the favorable peptide structure and aromatic residues arrangement for each system are shown as insets.

Secondary structure analysis was performed to investigate the effects of the nanomaterial curvature on the peptide's conformation. The STRuctural IDEntification (STRIDE) [53] algorithm was utilized to classify the peptide's secondary structure as a function of time. Secondary structure evolution plots depicting typical conformational trends exhibited by apoC-II(60-70) in the presence of C60, nanotube and graphene are shown in Figure 2. The initial 20 ns of simulation (equilibration) are also shown to highlight the conformational changes in the peptide induced by adsorption onto the nanomaterial surface.

The results show a structural transformation of the peptide upon adsorption to the nanomaterial surface. ApoC-II(60-70) in the presence of C60 was observed to curve around the particle, with a turn region around Gly65, as shown in the picture inset of Figure 2a. This structure allows for a large number of contacts to be made with the nanoparticle, dominated by π-interactions between the aromatic residues (Tyr63 and Phe67) and the C60 surface. Due to the presence of C60, apoC-II(60-70) is unable to form the inherent β-hairpin conformation [34], [37]. In one out of six simulations the peptide was able to dissociate within 10 ns of contact with the C60 particle. This suggests that the peptide can be weakly bound to the surface of the nanoparticle. Upon desorption apoC-II(60-70) was able to form a β-hairpin conformation (picture inset of Figure 2b). We note that the β-hairpin structure was found favorable for monomeric apoC-II(60-70) peptide in solution, and identified as an intermediate state on-pathway for fibril formation [34], [37], [75]. The results show that the presence of C60 inhibits the formation of the characteristic fibril favoring β-hairpin as well as the extended conformation suggesting that the interactions with the C60 may contribute to an increase in mobility (see Figure S1) and facilitate the formation of fibril incompetent conformations. Recent work by Andujar et al. where they showed that C60 induced significant destabilization of the amyloid-β fibrils by disrupting the hydrophobic contacts and salt-bridges between the β-sheets [76] is in line with our work. This suggests that C60 can be used as a prototype for the design of potential fibril inhibitors.

The secondary structure evolution plot of apoC-II(60-70) in the presence of a nanotube shows that the peptide exhibits different structural features compared to those in the presence of C60. The peptide tends to elongate across the surface of the nanotube, while adopting mostly turn and coil motifs. This behavior is a result of the large surface area available for contact on the nanotube (Figure 2c). The strong affinity between the nanotube and peptide is assisted by π-π interactions between the aromatic rings of the peptide and the nanotube. The curvature of the nanotube enables the peptide to arch, which facilitates the short-lived formation of a hydrogen bond between Ser61 and Thr64. In comparison to the C60 simulations, the peptide was less dynamic on the surface of the nanotube, as seen from the smaller number of conformations sampled by the peptide following the adsorption and immobilization on the nanotube surface (Figure 2c and Figure S1).

The simulations of apoC-II(60-70) in the presence of graphene exhibited similar structural features to those seen in the presence of the nanotube. Upon adsorption, the peptide elongates along the graphene surface and features predominantly turn and coil structures (Figure 2d). The large surface area available for interactions enables the peptide to freely slide on the surface, while the favorable π-π stacking interactions between the aromatic residues of the peptide and the surface define its conformational features. Linse et al. showed extended nanoparticles enhance the probability of appearance of a critical nucleus for nucleation of protein fibrils, albeit for a different combination of nanomaterials and peptides [9]. This feature was determined as fibril-favoring in our previous works on apoC-II(60-70) oligomers [34], [37]. Other studies have also shown that carbon nanotubes and graphene surfaces facilitate a change in the conformation of peptides [30], [77] and π-stacking is an efficient mode of biological recognition of π-electron-rich carbon nanoparticles [23], [30]–[32], [52], [77], [78].

A common feature in all secondary structure plots is the presence of a persistent coil motif at the C-terminal end of the peptide, where predominantly hydrophilic residues reside. This suggests that the inherent preference for interaction with the polar environment by these residues is suppressed by the attractive van der Waals forces between the large surfaces presented by the nanomaterial and the peptide, preventing the peptide dissociation from the nanomaterial. Strong hydrophobic interactions between the WW domains and carbon nanotubes have also been associated with protein function “poisoning” and disruption of the protein active site [79].

Overall, it should be noted that the adsorbed peptide may adopt both fibril initiating as well as fibril incompetent conformations. However, our analyses indicate that the extended conformation adopted on the extended nanosurfaces is in line with the fibril competent structures we found through our previous modeling and experimental studies [33], [34], [37], [38], [49], [50]. In contrast the mobility and lack of secondary structure elements needed for the fibril formation by the C60 adsorbed peptide suggests the inhibiting role of this nanoparticle in the fibril formation.

**Figure 3:**
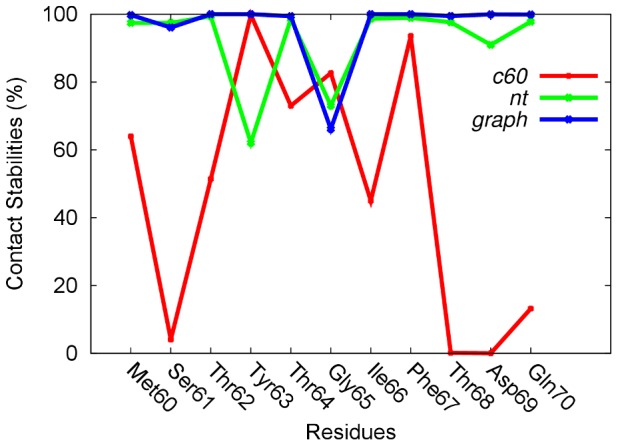
Persistent contacts. Contact stability plot of apoC-II adsorbed to C60 (red), carbon nanotubes (green) and graphene (blue).

To gain a more detailed understanding of the interactions involved in adsorption of apoC-II(60-70), the contact stabilities of the peptide's residues with each nanomaterial were investigated (Figure 3). Contact stabilities were calculated as the percentage of simulation time during which a contact was maintained between each residue and the respective nanomaterial. A contact was counted when the distance between a pair of atoms was less than 4 Å, which enabled us to account for van der Waals interactions between the peptide and the nanoparticle.

High contact stabilities were found for both the aromatic tyrosine (Tyr63) and phenylalanine (Phe67) residues with all nanomaterials. The stable π-stacking arrangements between the aromatic rings of the peptide and the electron-rich carbon rings of the surface, suggest that these are the key residues that contribute to the strong interactions between the peptide and the carbonaceous nanomaterials, in line with other studies [23], [30]–[32], [52], [77], [78].

This effect is evident in all simulations, however in the C60 complex the aromatic residues dominate the interactions between the peptide and C60 surface, while the other residues exhibit less persistent contacts. Interestingly, Tyr63 exhibits higher binding affinity to C60 compared to Phe67, in accordance with a DFT study that showed Phe and Tyr bind with a similar strength to the nanotube, while Tyr exhibits a stronger binding to C60 [80], [81]. In contrast, the large contact area presented by the carbon nanotubes and graphene results in higher contact stabilities with all (not just the aromatic) residues, this effect being most evident on the graphene surface.

In our recent studies we showed that the orientation of the aromatic side chains is different in the fibril-forming and fibril-inhibiting arrangements [34], [37]. The simulations of apoC-II(60-70) in the presence of C60 exhibited structures where the aromatic rings were positioned on the same side of the peptide which enhance the π-stacking interactions with the small, highly curved C60 particle. This ring arrangement was postulated to inhibit fibril formation [34], [37]. In contrast, the aromatic rings did not show a specific facial preference in the nanotube and graphene complex simulations (Figure 2c,d). This was due to the large contact area and stronger hydrophobic interactions presented by these materials, which formed the aromatic ring stacking upon adsorption of the peptide.

**Figure 4:**
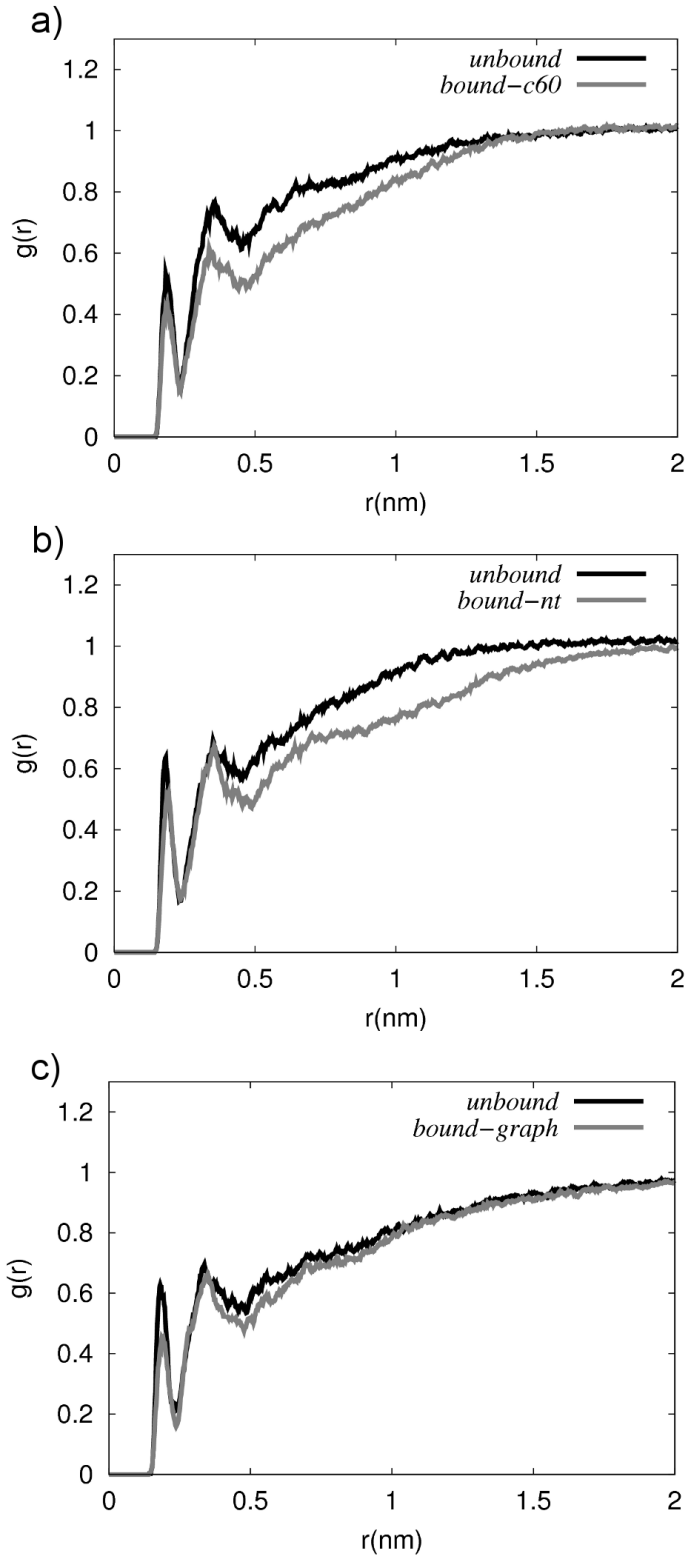
Radial distribution functions of water. RDFs of the water probability density, *g(r)*, as a function of the distance, *r*, of water (O) from the side chain (H) atoms of apoC-II(60-70). RDFs for the peptide in a free (unbound) and adsorbed (bound) to a) C60, b) nanotube and c) graphene are shown.

A series of radial distribution functions (RDF) were calculated to determine the degree of water structuring around the peptide in solution and when bound to the nanomaterial surface. The results provide an insight into the extent of desolvation of the peptide conformation upon binding to the different nanomaterials. Typical RDFs of the peptide side-chain hydrogen atoms (H) with respect to the water oxygen atoms (O) are shown in Figure 4. For all systems, the RDF profiles show a peak at ∼2 Å, representing the first hydration shell, indicating hydrogen bonding between water and the apoC-II(60-70) side-chains. The results also show the presence of a second hydration shell at ∼4 Å. The RDFs of peptide-nanomaterial complexes exhibit an attenuation of the overall probability density, suggesting the exclusion of water due to the hydrophobic contact between the peptide and nanoparticle manifests in the RDFs through the lowering of the occurrence of water at larger separation distances in the bound state. Indeed, desolvation effects have been shown to be favorable in the self-assembly of cyclic peptides on carbon nanotubes [31].

### Peptide-nanomaterial dissociation free energies in solution

**Figure 5:**
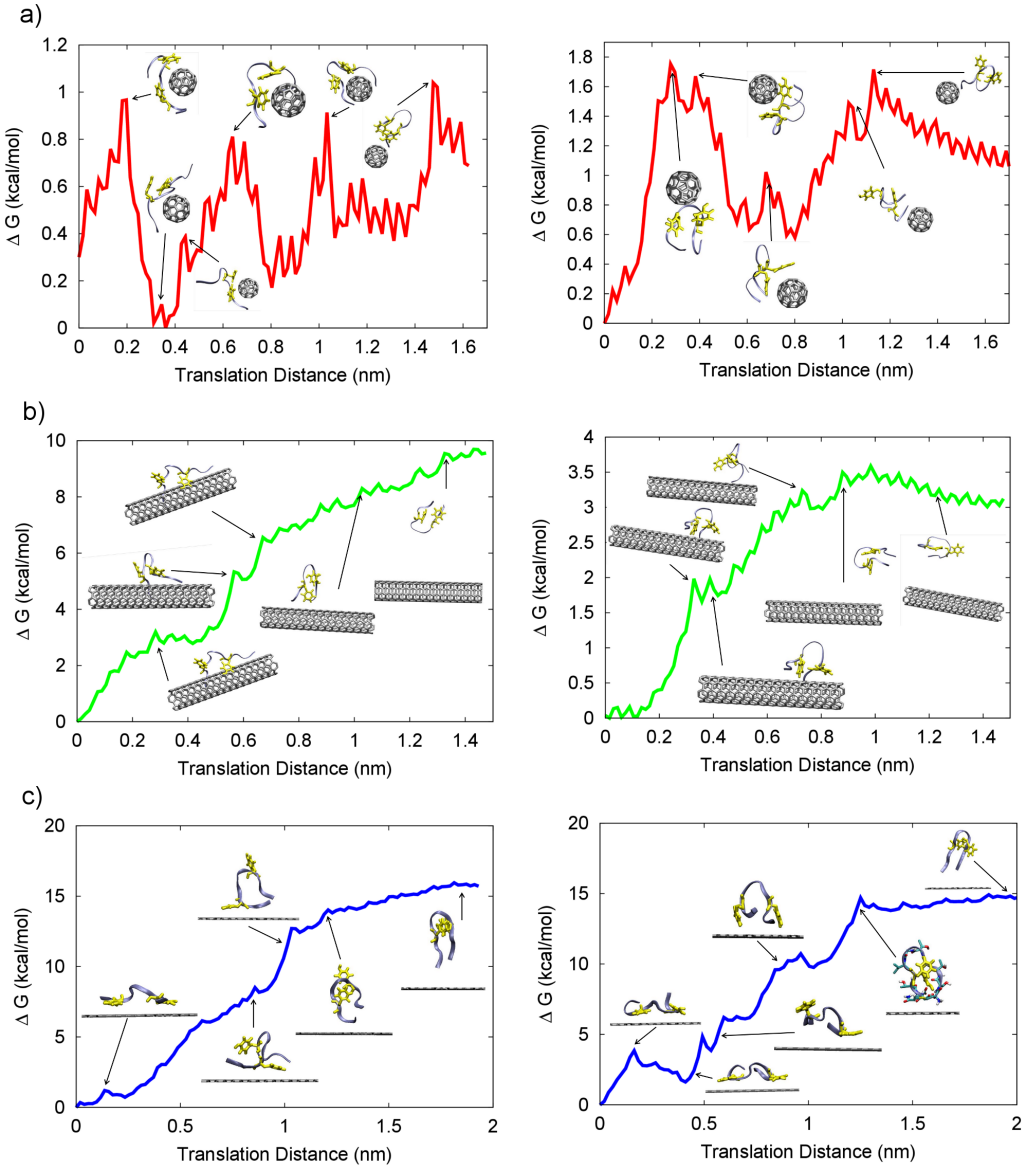
Peptide-nanomaterial free energy of dissociation. The free energy of dissociation of apoC-II(60-70) peptide from a) C60 (red); b) nanotube (green) and c) graphene (blue) surface. Different initial conformations of the peptide on the nanomaterials surface were examined to improve the conformational sampling. Screenshots depicting typical representative structures of important events on the free energy surface are shown as insets. For clarity the peptide structure is drawn as ribbon (iceblue) and the aromatic residues are shown as licorice (yellow).

To characterize the binding of apoC-II(60-70) peptide to each nanomaterial in the presence of solvent, the free energy of dissociation was calculated using umbrella sampling (potential of mean force, PMF) together with the weighted histogram analysis method (WHAM) [60]. This approach is applied to explicitly solvated systems and accounts for both the enthalpic and entropic contributions to the dissociation free energies. Two bound equilibrium complex structures were studied for each system to enhance sampling. The PMFs detailing the dissociation pathway of apoC-II(60-70) from each nanomaterial are presented in Figure 5.

The results demonstrate that higher degree of curvature reduces the surface area available for adsorption, and the dissociation free energy indicates that binding to C60 is weakest and binding to graphene is strongest of the systems investigated. Here, a lower value indicates a weaker binding. The size of the peptide does not allow for complete wrapping of the C60, therefore in this complex the peptide is quite mobile with terminal residues remaining free and not forming close contacts with the nanoparticle, as can be seen in the residue contact stability plot in Figure 3. Figure 5a indicates that the dissociation energy is dependent on the adsorbed peptide conformation. This suggests that C60 induces significant structural lability in apoC-II(60-70) preventing it from adopting stable conformations, in line with the peptide evolution observed through molecular dynamics trajectories (Figure 2 and Figure S1). A higher free energy of dissociation (∼1.8 kcal/mol) was obtained for the peptide that had a larger number of contacts with C60 and whose aromatic rings were continuously interacting with the C60 particle. In contrast, the system where the two aromatic rings of the peptide predominantly formed π-stacking between themselves rather than with the nanoparticle resulted in a lower dissociation energy (∼1.1 kcal/mol). The peaks and troughs are caused mostly by the transient π-stacking interactions, with peaks observed when contacts are broken, as illustrated by the insets in Figure 5. In our previous work on apoC-II(60-70) we showed that an increase in conformational flexibility and dynamics can slow down or even inhibit fibril formation [34], therefore it appears that interactions with the C60 can induce a similar, fibril inhibiting, effect.

We note that generally PMF plots for the C60-peptide system are noisier than those for the nanotube and graphene systems which is due to the transient nature of the contacts and increased mobility of apoC-II(60-70) when in contact with C60, rather than due to insufficient conformational sampling. The effect was verified by continuing the umbrella sampling simulations for a further 15 ns per window and observing that the resultant PMFs did not show significant differences (figures not shown). Similarly, the free energy differences seen between the multiple simulations of the peptide-nanotube system are due to the variety of structures sampled along each dissociation pathway. As expected, the predominantly elongated peptide conformation (Figure 5b, left) enabled a larger number of contacts between the peptide and the nanotube, which resulted in a higher free energy of dissociation (∼8.6 kcal/mol). The peptide exhibiting mostly coiled structures made fewer contacts with the nanotube (Figure 5b, right) which in turn required less energy (∼3.5 kcal/mol) to dissociate from it. The smoother PMF plots for the peptide-nanotube systems are a result of the persistent interactions between the components, in keeping with the results of our classical MD simulations.

The PMF plots representing the dissociation free energy of apoC-II(60-70) from graphene exhibited conformation independent pathways. As seen from the MD results, the π-stacking between apoC-II(60-70) and graphene contributes to the formation of elongated peptide structures and restricts the conformational flexibility of the peptide. Repeat simulations resulted in dissociation free energies of ∼15 kcal/mol irrespective of the conformations sampled along the dissociation pathway.

We note that dissociation energy peaks occur when a large number of contacts are broken, such as during the illustrated dislocation of the aromatic residues from the graphene surface (see insets of Figure 5c). In contrast, as the peptide is slowly pulled away from the surface a characteristic smooth dissociation energy profile is observed.

### Aromatic stacking and in vacuo binding energies

In addition to the classical simulation-derived dissociation free energies discussed above, we have used electronic structure calculations based on DFT to calculate *in vacuo* binding energies of selected frames derived from classical all-atom simulations. To investigate the role of aromatic residues (Tyr63 and Phe67) in driving the adsorption of apoC-II(60-70) onto carbon based nanomaterials, we developed an algorithm capable of tracking the position and orientation of the phenyl rings of the aromatic amino acids with respect to the aromatic rings of the nanomaterials' surface at every step of the MD trajectories (exemplar result shown in Supporting Information).

**Figure 6:**
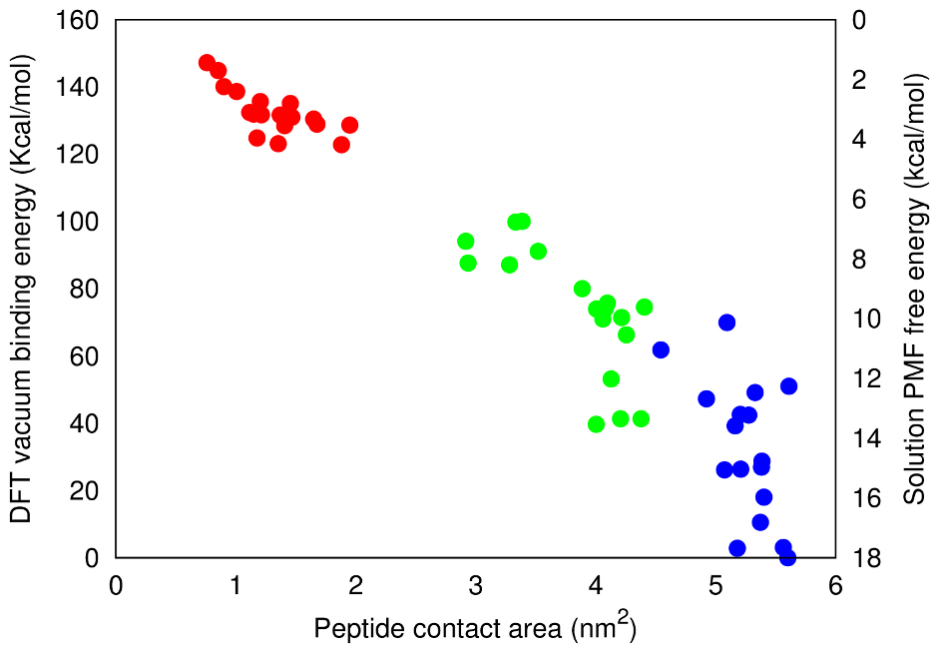
In vacuo binding energies. Relative DFT vacuum binding energies of apoC-II(60-70) adsorbed to C60 (red), carbon nanotube (green) and graphene (blue) vs the total contact area between the peptide and nanomaterial surface. The solution PMF free energy range for each nanomaterial is also shown (right axis, higher energy = stronger binding) to illustrate the correlation in energies between the classical and electronic structure methods.

The *in vacuo* binding energy of three representative frames from each π-stacking arrangement was obtained by DFT calculations using the ONETEP linear-scaling code [62], comprising a total of eighteen typical structures per peptide-nanoparticle complex. This analysis provides a measure of the relative binding of apoC-II(60-70) to a nanomaterial surface with respect to the contact area. The binding energy differences between the representative structures for each π-stacking configuration versus the peptide-nanomaterial contact area are shown in Figure 6 (tabulated form available in Supporting Information). The vacuum binding energies are shown relatively to the strongest bound state (apoC-II(60-70) on graphene). In this case, higher values indicate a weaker binding (left y-axis, Figure 6).

The *in vacuo* binding energy results confirm the trends observed in our explicitly solvated PMF free energies showing the strength of binding between apoC-II(60-70) and the nanomaterials to follow: C60<nanotube<graphene. In all systems the aromatic rings act like “anchors“ for binding the peptide to the carbon nanomaterials via π-π interactions.

DFT binding energy calculations confirm the finding from classical MD that apoC-II(60-70) exhibits strongest binding on graphene with a face-to-face π-stacking arrangement made by the two aromatic rings of the peptide and the surface. The all-atom MD simulations show that the flat graphene surface promoted sliding of the peptide (see Figure S2 in Supporting Information) and backbone elongation to optimize the π-stacking arrangement between the aromatic rings of the peptide and the substrate. This contributes to the peptide-graphene system having the largest aromatic and total contact area, which results in the strongest binding. The DFT binding energy also confirmed that apoC-II(60-70) exhibits a weaker binding to the nanotube and the weakest binding to C60, attributed to the increased nanosurface curvature which stimulates the formation of turns and loops in apoC-II(60-70) leading to a lower contact area between the peptide and nanoparticle. We note that C60 comprises both hexagonal and pentagonal carbon rings and, therefore, has a lower probability of face-to-face π-stacking with the six-membered aromatic rings of the peptide (statistical data shown in Supporting Information). This provides a further explanation for the significantly smaller contact area and weaker binding obtained for the peptide and C60 nanoparticle, compared to the nanotube and graphene systems (Figure 6 and Table S3).

**Figure 7:**
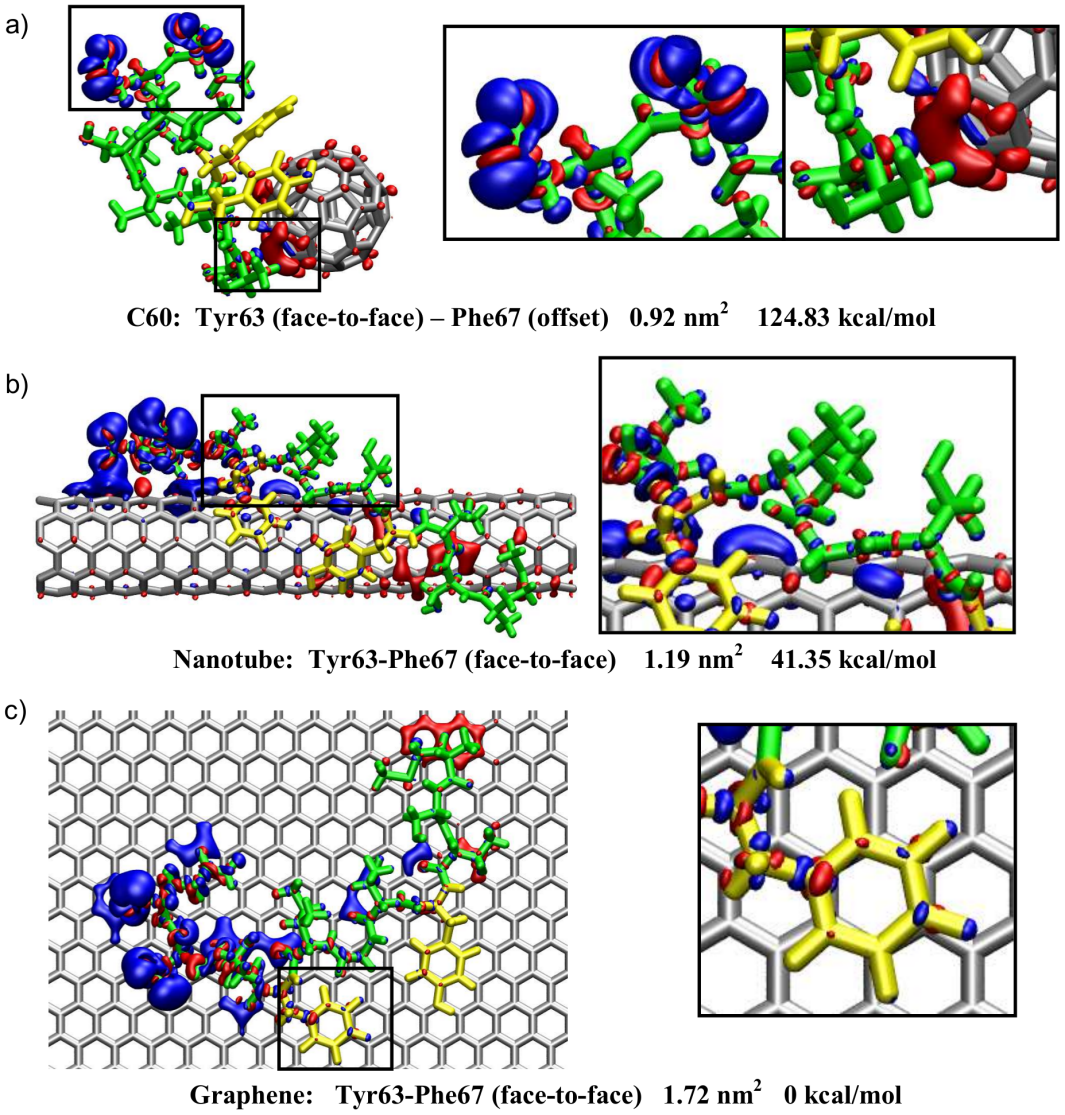
Electron density difference maps. Electron density difference maps of representative frames from the clustering analysis shown by an isosurface with isovalues of Δρ = +0.005e/Å^3^ and −0.005e/Å^3^. Red represents charge accumulation, and blue represents charge depletion. The aromatic rings are colored yellow for clarity. The respective structures' π-stacking arrangement, aromatic contact area and binding energy differences relative to the strongest bound state (face-to-face π arrangement on graphene, figure c) are also shown together with close-up insets of specific features to aid interpretations of the results for a) C60, b) nanotube and c) graphene.

Furthermore, using our DFT calculations we were able to examine the intra-peptide electrostatic interactions which play a significant role in determining the peptide's secondary structure and consequently the binding affinity to other materials. Electron density difference (Δρ) maps showing charge accumulation (red) and depletion (blue) upon peptide adsorption on each nanomaterial are presented in Figure 7. We can see that intra-peptide interactions are more significant in the proximity of nanoparticles with high curvature which have a reduced nanoparticle-peptide contact surface area, as Figures 6 and 7 demonstrate.

The greater surface area available on the flatter “hexagonal-only” surface of graphene allows for a more efficient π-stacking and a stronger peptide binding as shown in Figures 7c. Moreover, surface adsorbed elongated peptide conformations enable polar residues such as Thr, Ser and Gln to become more solvent exposed, thus exhibiting the “snorkeling effect” [82], [83] (see inset of Figure 7b), where the hydrophobic backbone interacts with the graphitic surface, while the polar side chains are protruding to the solvent. This lowers the overall contact area between the peptide and nanomaterial, and ultimately reduces the binding affinity. Figure 7 shows small electron density differences between the aromatic groups and the graphitic surfaces. This is in agreement with the study of Poenitzsch et al. where they observed weak charge-transfer interactions between aromatic groups and carbon nanotubes using scanning tunneling spectroscopy and Raman experiments [84]. Our electron density analysis shows that, generally, a weaker binding is a result of inefficient π-stacking arrangements and intra-peptide electrostatic interactions that reduce the peptide-surface interactions, as Table S3 demonstrates. Charge redistribution can also be seen between the peptide and nanoparticle surface (Figure 7), suggesting some polarizability effects occur between the peptide and nanomaterial. Specifically, a charge depletion can be seen at Asp69 and Gln70 in all systems, while a charge accumulation develops at the closely interacting sites of the peptide and nanomaterial surface. Figure 7a shows charge accumulation at Gly65 for the C60 complex, while the nanotube and graphene exhibit charge buildup in close proximity to the predominantly hydrophilic N-terminal region of the peptide (Met60, Ser61 and Thr62).

We note that the agreement between our classical simulations and the DFT studies suggest that the classical forcefield potentials employed here are able to capture the polarization effects inherent to peptide-nanoparticle systems. Moreover, a recent study using the dispersion corrected DFTB-D method, showed that although molecular mechanics techniques with fixed-charge forcefields do not explicitly incorporate polarizability, they can predict the strength of π-π interactions between aromatic moieties and carbon nanotubes [75]. This demonstrates that molecular dynamics simulations utilizing fixed charge forcefields provide a reasonable representation of the interactions between peptides and graphitic surfaces.

### Conclusions

Using classical forcefield and electronic structure calculations, we have shown that an amyloidogenic apoC-II(60-70) peptide exhibits a strong affinity for graphitic nanomaterials where binding is facilitated through π-π interactions between the aromatic residues of the peptide and the surface of the nanomaterial. This is generally achieved by the exclusion of water molecules from the peptide-nanomaterial interface.

The proximity of the C60 fullerene contributed to an increase in conformational lability of apoC-II(60-70), which was shown to prevent it from adopting fibril-favoring structural features. This finding is in line with the previous studies of oxidized apoC-II(60-70), where increased structural flexibility and dynamics were the key factors prohibiting this peptide to form fibrils, confirmed experimentally. Conversely, our data showed that the less curved nanotube and flat graphene nanomaterials promote elongated peptide conformations previously shown to form fibril seeds, which confirms recent findings that extended carbon nanosurfaces can act as templates able to encourage peptide fibril formation and growth.

Electronic binding energy and solution free energy calculations showed the binding affinity of apoC-II(60-70) was weakest for the C60 particle, followed by the nanotube, and strongest for the graphene. In all simulations these trends are due to the larger contact area available for peptide adsorption to the flatter graphene and nanotube than the highly curved C60. The increased curvature also results in reduced efficiency of aromatic π-stacking and higher intra-peptide electrostatic interactions which contributes to its weaker binding to the nanomaterials. The electronic structure calculations show that dimensionality that determines the electronic properties of the nanoparticle as well as size and curvature play a significant role in the contact area and binding mechanisms of the peptide. At the same time the intra-peptide interactions determined by the peptide sequence (i.e. presence of aromatic, aliphatic, polar/apolar amino acids) affect the binding mechanism of peptides to nanoparticles. The observed agreement between the classical and electronic structure calculations show that molecular dynamics simulations utilizing fixed charge forcefields provide reasonable representation of the interactions between peptides and graphitic surfaces.

In summary, our results highlight that hydrophobic nanoparticles have multiple notable effects on the peptide structure, dynamics and binding affinity. We have demonstrated that dimensionality and different degree of curvature can either facilitate or hinder the interaction of amyloidogenic peptides with the nanosurfaces and make them adopt conformations capable of inhibiting or promoting fibril development, as shown in our recent experiments. These findings can be important for rational design of amyloid fibril inhibitors as well as for clarification of possible toxic effects of carbon based nanomaterials.

## Supporting Information

Figure S1 **Peptide atomic position fluctuation in the presence of each nanoparticle.** Root mean square fluctuation of the atomic positions in each residue in the presence of C60 (red), nanotube (green) and graphene (blue).
(TIFF)

Figure S2 **Exemplar plot depicting the output from the aromatic arrangement tracking analysis.** Aromatic tracking results showing the position of the center of mass of the apoC-II(60-70) with respect to the number of aromatic contacts (face-to-face and offset π-stacking) occurring at this position.
(TIFF)

Table S1 **Starting orientations and arrangements of wild-type apoC-II(60-70) peptide.** Starting orientations of the wild-type apoC-II(60-70) peptide relative to each nanoparticle. The system names and total simulation time are also shown.
(TIFF)

Table S2 **Starting orientations and arrangements of helical apoC-II(60-70) peptide.** Starting orientations of the helical apoC-II(60-70) peptide relative to each nanoparticle. The system names and total simulation time are also shown.
(TIFF)

Table S3 **Tabulated in vacuo binding energy results.** *In vacuo* binding energies (relative to the strongest bound complex, face-to-face π-stacking on graphene), *and* absolute binding energies together with total contact area of the representative frames for each arrangement category and nanomaterial. Structural comments for each frame are also included.
(DOC)

Table S4 **Aromatic arrangement occupancy.** The total frame occupancy (%) within each main aromatic arrangement category: no π-stacking (arrangement 1), offset π-stacking (arrangements 2–5) and face-to-face π-stacking (arrangement 6) occurring on c60, nanotube and graphene as determined from the aromatic tracking and cluster analysis.
(DOC)
