# Supplementary figures and images for: Dimensionality of Carbon Nanomaterials Determines the Binding and Dynamics of Amyloidogenic Peptides: Multiscale Theoretical Simulations
Source: PLoS Comput Biol. 2013 Dec 5;9(12):e1003360. doi: 10.1371/journal.pcbi.1003360 (PMC3854483; doi:10.1371/journal.pcbi.1003360)

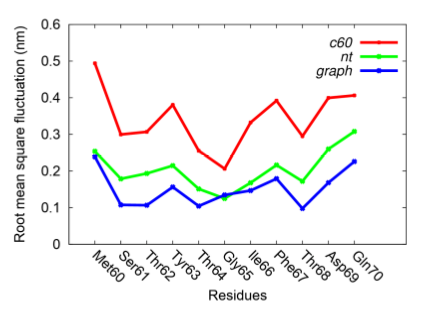

Supplement: Figure S1 — Peptide atomic position fluctuation in the presence of each nanoparticle. Root mean square fluctuation of the atomic positions in each residue in the presence of C60 (red), nanotube (green) and graphene (blue). (TIFF) [file pcbi.1003360.s001.tiff]

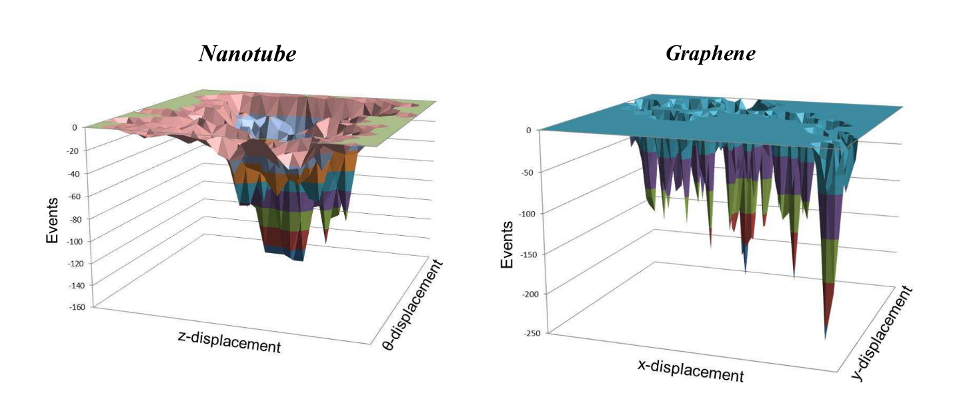

Supplement: Figure S2 — Exemplar plot depicting the output from the aromatic arrangement tracking analysis. Aromatic tracking results showing the position of the center of mass of the apoC-II(60-70) with respect to the number of aromatic contacts (face-to-face and offset π-stacking) occurring at this position. (TIFF) [file pcbi.1003360.s002.tiff]

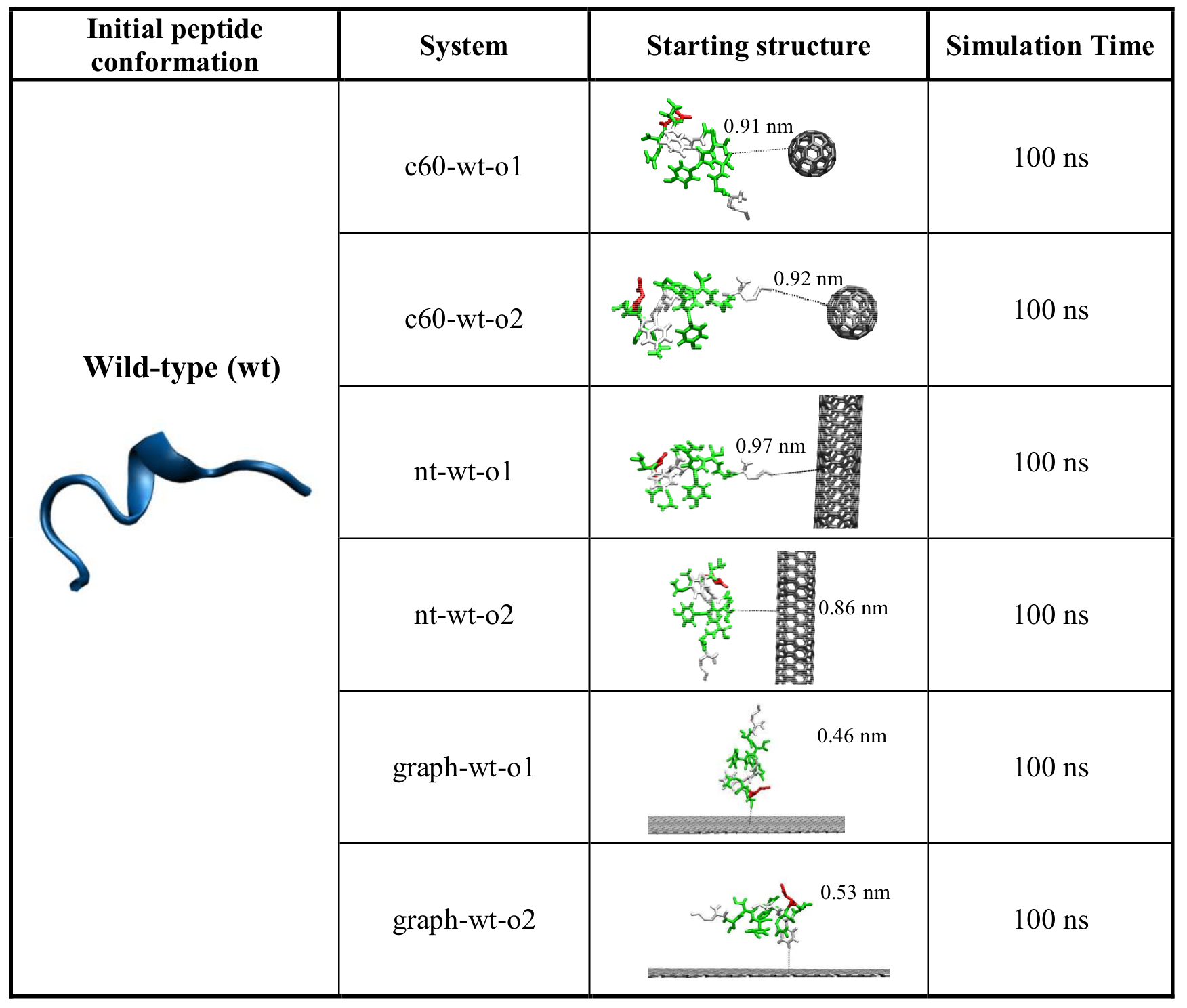

Supplement: Table S1 — Starting orientations and arrangements of wild-type apoC-II(60-70) peptide. Starting orientations of the wild-type apoC-II(60-70) peptide relative to each nanoparticle. The system names and total simulation time are also shown. (TIFF) [file pcbi.1003360.s003.tiff]

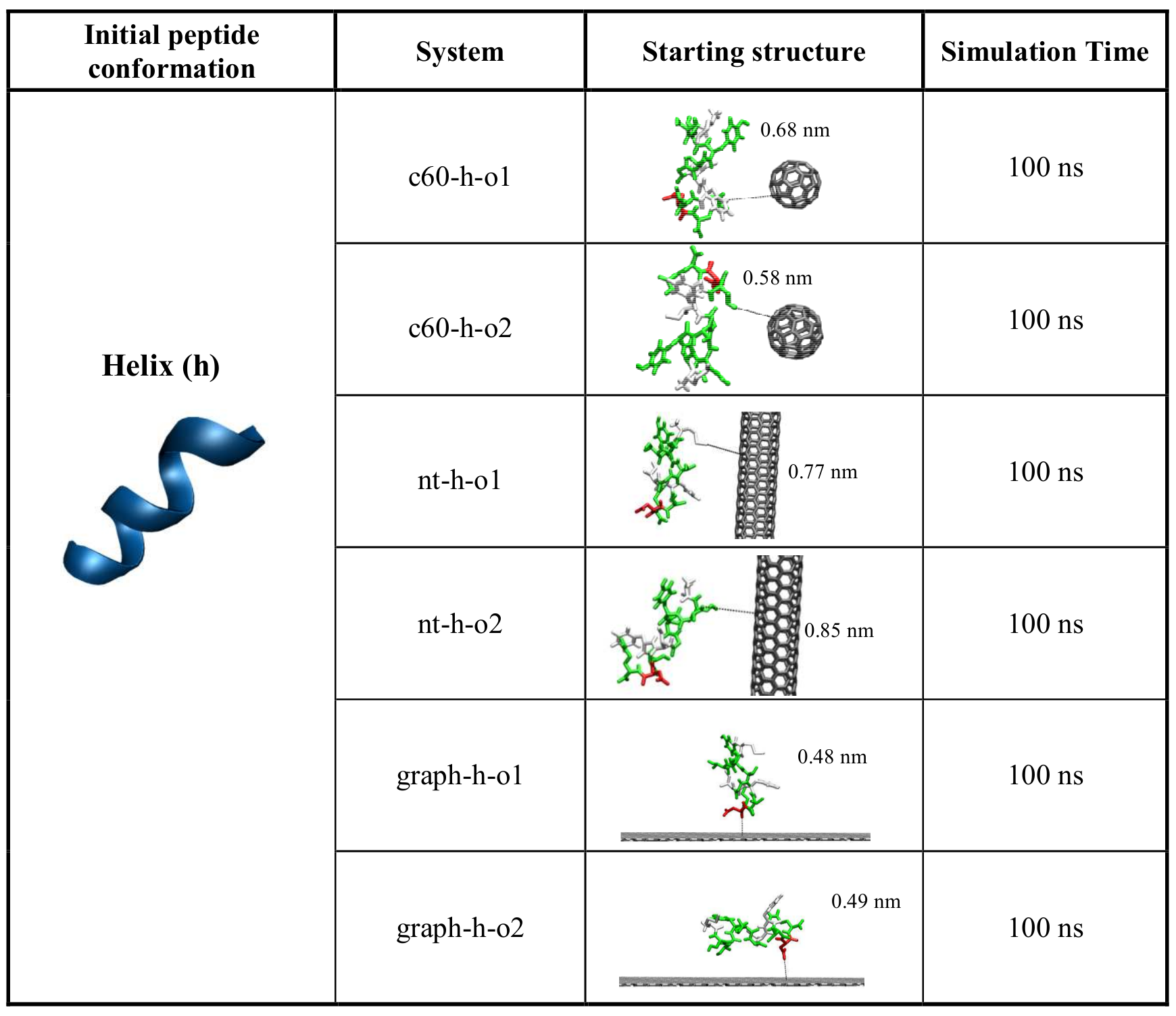

Supplement: Table S2 — Starting orientations and arrangements of helical apoC-II(60-70) peptide. Starting orientations of the helical apoC-II(60-70) peptide relative to each nanoparticle. The system names and total simulation time are also shown. (TIFF) [file pcbi.1003360.s004.tiff]
