# Supplementary material for: Dimensionality of Carbon Nanomaterials Determines the Binding and Dynamics of Amyloidogenic Peptides: Multiscale Theoretical Simulations
Source: PLoS Comput Biol. 2013 Dec 5;9(12):e1003360. doi: 10.1371/journal.pcbi.1003360 (PMC3854483; doi:10.1371/journal.pcbi.1003360)
Supplement: Table S3 — Tabulated in vacuo binding energy results. In vacuo binding energies (relative to the strongest bound complex, face-to-face π-stacking on graphene), and absolute binding energies together with total contact area of the representative frames for each arrangement category and nanomaterial. Structural comments for each frame are also included. (DOC) [file pcbi.1003360.s005.doc]

**Table S3**

| **C60** | | | | |
| --- | --- | --- | --- | --- |
| **Aromatic**  **Group** | **Relative Binding Energies (kcal/mol)** | **Absolute Binding Energies (kcal/mol)** | **Peptide Contact Area (nm^2^)** | **Comments** |
| 1 | 132.45 | -33.56 | 1.12 | Curved, terminal residues bound to C60 |
| 1 | 131.76 | -34.25 | 1.21 | Curved, terminal residues exposed to solvent |
| 1 | 135.67 | -30.34 | 1.21 | Curved, terminal residues exposed to solvent |
| 2 | 147.22 | -18.79 | 0.76 | Curved, terminal residues exposed to solvent |
| 2 | 140.13 | -25.88 | 0.90 | Curved, terminal residues exposed to solvent |
| 2 | 124.84 | -41.17 | 1.18 | Curved, terminal residues exposed to solvent |
| 3 | 131.68 | -34.33 | 1.37 | Curved, terminal residues bound to C60 |
| 3 | 130.44 | -35.57 | 1.65 | Curved, terminal residues exposed to solvent, substantial H-bonding |
| 3 | 131.04 | -34.97 | 1.47 | Curved, terminal residues bound to C60, some H-bonding |
| 4 | 123.14 | -42.87 | 1.35 | Curved, terminal residues bound to C60, some H-bonding |
| 4 | 131.94 | -34.07 | 1.15 | Curved, terminal residues bound to C60, some H-bonding |
| 4 | 135.09 | -30.92 | 1.46 | Curved, terminal residues bound to C60, some H-bonding |
| 5 | 122.85 | -43.16 | 1.88 | Curved, terminal residues exposed to solvent, substantial H-bonding |
| 5 | 128.50 | -37.51 | 1.41 | Curved, terminal residues bound to C60, some H-bonding |
| 5 | 144.89 | -21.12 | 0.85 | Curved, terminal residues exposed to solvent, substantial H-bonding |
| 6 | 128.66 | -37.35 | 1.95 | Curved, terminal residues exposed to solvent, substantial H-bonding |
| 6 | 128.96 | -37.05 | 1.68 | Curved, terminal residues exposed to solvent, substantial H-bonding |
| 6 | 138.66 | -27.35 | 1.01 | Curved, terminal residues bound to C60, substantial H-bonding |
| **Nanotube** | | | | |
| 1 | 91.14 | -74.87 | 3.52 | Curved, terminal residues bound to nanotube, substantial H-bonding |
| 1 | 99.86 | -66.15 | 3.33 | Curved, terminal residues bound to nanotube, substantial H-bonding |
| 1 | 100.05 | -65.96 | 3.39 | Curved, terminal residues bound to nanotube, substantial H-bonding |
| 2 | 94.12 | -71.89 | 2.92 | Curved, terminal residues bound to nanotube, substantial H-bonding |
| 2 | 71.01 | -95.00 | 4.06 | Elongated, terminal residues bound to nanotube |
| 2 | 75.73 | -90.28 | 4.10 | Elongated, terminal residues bound to nanotube |
| 3 | 80.02 | -85.99 | 3.89 | Elongated, terminal residues bound to nanotube, snorkelling effect |
| 3 | 74.00 | -92.01 | 4.01 | Elongated, terminal residues bound to nanotube, snorkelling effect |
| 3 | 74.18 | -91.83 | 4.08 | Elongated, terminal residues bound to nanotube, snorkelling effect |
| 4 | 87.65 | -78.36 | 2.94 | Curved, terminal residues bound to nanotube, substantial H-bonding |
| 4 | 39.70 | -126.31 | 4.01 | Elongated, terminal residues bound to nanotube, snorkeling effect |
| 4 | 71.44 | -94.57 | 4.22 |  |
| 5 | 41.35 | -124.66 | 4.38 | Elongated, terminal residues bound to nanotube, snorkeling effect |
| 5 | 74.58 | -91.43 | 4.41 | Curved, terminal residues bound to nanotube, substantial H-bonding |
| 5 | 66.34 | -99.67 | 4.26 | Elongated, terminal residues bound to nanotube, snorkeling effect occurring, some H-bonding |
| 6 | 53.20 | -112.81 | 4.13 | Elongated, terminal residues bound to nanotube, snorkeling effect |
| 6 | 87.13 | -78.88 | 3.28 | Curved, MET60 terminal residue exposed to solvent |
| 6 | 41.35 | -124.66 | 4.21 | Elongated, terminal residues bound to nanotube |
| **Graphene** | | | | |
| 1 | 47.28 | -118.73 | 4.92 | Curved, terminal residues bound to graphene, substantial H-bonding, snorkeling effect occurring |
| 1 | 42.64 | -123.37 | 5.21 | Curved, terminal residues bound to graphene, substantial H-bonding |
| 1 | 69.97 | -96.04 | 5.10 | Curved, terminal residues bound to graphene, substantial H-bonding |
| 2 | 49.14 | -116.87 | 5.33 | Curved, terminal residues bound to graphene, substantial H-bonding |
| 2 | 18.02 | -147.99 | 5.40 | Curved, terminal residues bound to graphene, substantial H-bonding |
| 2 | 26.15 | -139.86 | 5.08 | Curved, terminal residues bound to graphene, substantial H-bonding |
| 3 | 3.06 | -162.95 | 5.57 | Elongated, terminal residues bound to graphene |
| 3 | 2.81 | -163.20 | 5.18 | Elongated, terminal residues bound to graphene, snorkeling effect |
| 3 | 42.42 | -123.59 | 5.28 | Elongated, terminal residues bound to graphene, snorkeling effect |
| 4 | 51.00 | -115.01 | 5.61 | Curved, terminal residues bound to graphene, substantial H-bonding |
| 4 | 26.96 | -139.05 | 5.38 | Curved, terminal residues bound to graphene, substantial H-bonding |
| 4 | 28.72 | -137.29 | 5.39 | Curved, terminal residues bound to graphene, substantial H-bonding |
| 5 | 39.23 | -126.78 | 5.16 | Elongated, terminal residues bound to graphene, snorkeling effect |
| 5 | 10.55 | -155.46 | 5.37 | Elongated, terminal residues bound to graphene |
| 5 | 26.37 | -139.64 | 5.21 | Elongated, terminal residues bound to graphene |
| 6 | 61.85 | -104.16 | 4.54 | Elongated, terminal residues bound to graphene |
| 6 | 53.60 | -112.41 | 5.31 | Curved, terminal residues bound to graphene, substantial H-bonding |
| 6 | 0.00 | -166.01 | 5.60 | Elongated, MET60 and SER61 terminal residues exposed to solvent, snorkeling effect occurring |
