# Supplementary material for: Dimensionality of Carbon Nanomaterials Determines the Binding and Dynamics of Amyloidogenic Peptides: Multiscale Theoretical Simulations
Source: PLoS Comput Biol. 2013 Dec 5;9(12):e1003360. doi: 10.1371/journal.pcbi.1003360 (PMC3854483; doi:10.1371/journal.pcbi.1003360)
Supplement: Table S4 — Aromatic arrangement occupancy. The total frame occupancy (%) within each main aromatic arrangement category: no π-stacking (arrangement 1), offset π-stacking (arrangements 2–5) and face-to-face π-stacking (arrangement 6) occurring on c60, nanotube and graphene as determined from the aromatic tracking and cluster analysis. (DOC) [file pcbi.1003360.s006.doc]

**Table S4**

| **Group** | **C60**  **(%)** | **Nanotube (%)** | **Graphene (%)** |
| --- | --- | --- | --- |
| No π-stacking | 77.1% | 28.7% | 12.9% |
| Offset π-stacking | 18.0% | 61.9% | 63.6% |
| Face-to-face π-stacking | 4.9% | 9.4% | 23.5% |
